# Cerebrospinal fluid glutamate changes in functional movement disorders

**DOI:** 10.1038/s41531-020-00140-z

**Published:** 2020-12-04

**Authors:** Benedetta Demartini, Roberto William Invernizzi, Laura Campiglio, Tommaso Bocci, Andrea D’Arrigo, Andrea Arighi, Francesca Sciacca, Daniela Galimberti, Elio Scarpini, Orsola Gambini, Alberto Priori

**Affiliations:** 1 Dipartimento di Scienze della Salute, Università degli Studi di Milano, Milano, Italy; 2 Unità di Psichiatria II, A.O. San Paolo, ASST Santi Paolo e Carlo, Milano, Italy; 3 “Aldo Ravelli” Research Center for Neurotechnology and Experimental Brain Therapeutics, Università degli Studi di Milano, Milano, Italy; 4 Laboratorio Neurochimica e Comportamento, Istituto di Ricerche Farmacologiche “Mario Negri” IRCCS, Milano, Italy; 5 III Clinica Neurologica, A.O. San Paolo, ASST Santi Paolo e Carlo, Milano, Italy; 6 UOSD Neurologia, Malattie Neurodegenerative, Fondazione IRCCS Ca’ Granda Ospedale Maggiore Policlinico, Milano, Italy; 7 Dipartimento Biochimica Specialistica Neurologica e Neurofarmacologia, Fondazione IRCCS Istituto Neurologico Carlo Besta, Milano, Italy; 8 Università degli Studi di Milano, Centro Dino Ferrari, Research Center for the Study of Molecular Mechanisms of Neuro-Psycho-Geriatric Diseases, Milano, Italy

**Keywords:** Neurodegeneration, Diagnostic markers

## Abstract

The aim of this study was to assess cerebrospinal fluid (CSF) concentrations of specific amino acids using a high-performance liquid chromatography system in a sample of patients with functional movement disorders (FMDs) and in a sample of controls. CSF levels of glutamate were significantly lower in patients with FMD than in controls. This finding argues in favor of glutamatergic dysfunction in the pathophysiology of FMD.

Glutamate has an important role as the primary excitatory neurotransmitter in the central nervous system^1^. Growing evidence suggests that abnormalities in glutamatergic neurotransmission, via the *N*-methyl-d-aspartate receptor (NMDA-R), have a key role in the pathophysiology of several neuropsychiatric conditions, such as schizophrenia, mood disorders, and Alzheimer’s disease^2–4^. Recently, a possible role of glutamate + glutamine (Glx) has been suggested in the pathophysiology of FMDs (also called conversion disorders), a neuropsychiatric condition characterized by the presence of motor symptoms that cannot be explained by typical neurological diseases or other medical conditions^5^. Although they are highly prevalent and have a considerable impact on national health systems, the pathophysiology of FMD remains unclear. In the past decade, new hypotheses based on the integration between psychology and neurobiology have been formulated, gradually shifting the attention of the scientific community from traditional psychoanalytic theories to models considering neurobiological factors^6^. Functional neuroimaging studies have underlined abnormalities in FMD at the level of brain network activity, connectivity, and specific anatomic areas of altered metabolic demand during tasks (mainly the limbic system)^7^. With respect to Glx, Demartini et al.^5^ recently showed, through a brain magnetic resonance spectroscopy (MRS) technique, that patients with FMD presented significantly higher levels of Glx in the limbic system than healthy controls. The authors hypothesized that abnormal increase in Glx in the limbic system might have a central pathophysiological role in FMD onset and maintenance, possibly by altering limbic–motor interactions. However, no studies have been conducted assessing the levels of glutamate and glutamine in the cerebrospinal fluid (CSF) of patients with FMD.

Given the evidence of a potential role of Glx in the pathophysiology of FMD, we aimed to retrospectively assess CSF levels of glutamate and glutamine in a sample of patients with FMD and in a sample of controls. Moreover, we also assessed the CSF levels of two other amino acids, alanine and asparagine, serving as controls.

Eight patients with FMD and nine controls were recruited. The Kolmogorov–Smirnov test showed that all the continuous variables respected the assumption of a normal distribution (all *p* > 0.05).

Patients and controls did not differ with respect to gender, age, or time elapsed between the date of the lumbar puncture (LP) and date of CSF amino acid measurements (Table 1). No major psychiatric comorbidity was found in the whole sample. No significant correlations were found between amino acid levels and (i) severity of FMD or (ii) duration of disease (all *p* > 0.05). Multivariate analysis of variance (ANOVA) showed that CSF levels of glutamate were significantly lower in patients with FMD (mean 1.23 ± 0.66) than in controls (mean 2.17 ± 1.05) (*F* (1,15) = 4.751, *p* = 0.046). Neither CSF levels of glutamine (mean 341.25 ± 87.01 for patients with FMD, mean 340.99 ± 65.57 for controls), asparagine (mean 4.52 ± 2.60 for patients with FMD, mean 5.02 ± 0.97 for controls), nor alanine (mean 30.35 ± 10.03 for patients with FMD, mean 31.26 ± 8.47 for controls) differed between the two groups (*F* (1,15) = 0.000, *p* = 0.994 for glutamine, *F* (1,15) = 0.293, *p* = 0.596 for asparagine, *F* (1,15) = 0.041, *p* = 0.841 for alanine) (Fig. 1).

**Table 1:** Demographic variables of patients with functional movement disorders and controls.

	Patients with FMD (*N* = 8)	Controls (*N* = 9)	Significance
Gender, female, *n* (%)	8 (100)	7 (77.8)	*X*^2^ = 2.015, *p* = 0.471
Age (years), mean (SD)	47.13 (16.93)	42.00 (17.41)	*t* = −0 to 614, *p* = 0.549
PMDRS score, mean (SD)	6.2 (3.8)		
Time elapsed between date of the lumbar puncture and date of CSF amino acids measurements (months), mean (SD)	10.50 (10.57)	11.78 (4.02)	*t* = 0.337, *p* = 0.741
Time elapsed between onset of symptoms and date of lumbar puncture (months), mean (SD)	5.6 (3.2)	4.6 (2.6)	*t* = 0.244, *p* = 0.543

*FMD* functional movement disorders, *SD* standard deviation, *CSF* cerebrospinal fluid.

**Fig. 1:**
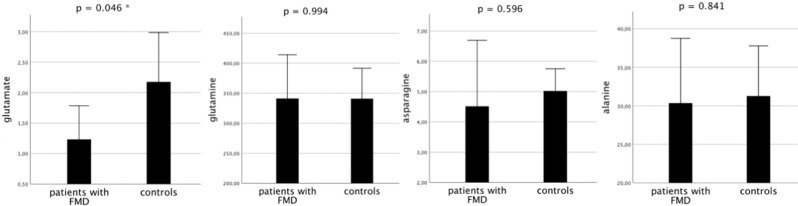
Levels of amino acids in the CSF of patients with FMD and controls. CSF levels of glutamate were significantly lower in patients with FMD than in healthy controls. CSF levels of glutamine, asparagine, and alanine in patients with FMD were not different from those of controls. Amino acid levels are expressed in μmol/L. Error bars represent standard errors.

In this study, which was designed to provide information on CSF glutamate and glutamine and subsequently on pathophysiological mechanisms in FMD, the main finding is that patients with FMD have decreased CSF glutamate compared to a group of controls. To the best of our knowledge, this is the first study exploring the levels of glutamate and glutamine in the CSF of patients with FMD. Previous studies have assessed CSF glutamate and glutamine in different neuropsychiatric conditions, with contradictory results: some studies found glutamate or glutamine to be decreased in patients’ CSF, while others found glutamate or glutamine to be increased^2–4^. The discrepancy in CSF glutamate and glutamine levels observed in previous studies might be due to different analytical methods used. Another reason for the inconsistency of results may be a time-dependent in vitro hydrolysis of glutamine to glutamate in the CSF^8^. In the present study, the mean storage time of patients with FMD and control CSF samples was very similar (mean 13 ± 11 and 14 ± 10 months, respectively). In addition, no significant differences in CSF glutamine levels were observed between patients with FMD and control samples. Thus, glutamine hydrolysis, if present, unlikely contributed to differences in glutamate levels observed in the present study. However, previous studies suggested an altered glutamatergic function as a possible key feature in the pathophysiology of different neuropsychiatric disorders, including schizophrenia, depression, and Alzheimer’s disease^2–4^. Although an indirect measure of glutamatergic activity, the reduction we found in CSF glutamate of patients with FMD suggests that hypofunction of the glutamatergic system may be present in these patients. It is well known that glutamate plays an important role in brain synaptic plasticity and is involved in several cognitive functions, such as learning and memory, in the hippocampus and neocortex. A proper glutamatergic tone is fundamental for neuronal differentiation and connection. Reduced glutamatergic neurotransmission is known to impair neuronal migration and circuitry maturation, ultimately leading to the development of neuropsychiatric disorders^9^. Although certain data on FMD are still not available in the literature, we hypothesize that dysregulation of the glutamatergic system might have a specific role in the pathophysiology of FMD. Our hypothesis also fits with the neurophysiological data showing an impairment of cortical excitatory networks, as proven by the well-known paradoxical decrease in motor excitability during motor imagery, which is not restricted to the clinically affected body part^10,11^. Moreover, given the absence of any major psychiatric comorbidity in our sample, which could have acted as a confounding factor, we might assume that the abnormal level of glutamate detected is specific for FMD. We did not find any significant correlation between CSF glutamate levels and (i) severity of FMD or (ii) duration of disease. This result, although possibly altered by the small sample size, might suggest that CSF glutamate levels are independent of the severity and duration of symptoms.

Our results might be in contrast with our previous spectroscopic finding according to which Glx in the limbic system of patients with FMD was increased with respect to healthy controls^5^. Nevertheless, first, it is still unclear whether CSF glutamate levels reflect the actual synaptic activity in each corticolimbic region; CSF levels may possibly represent the overall equilibrium of the extraneuronal space^12^. Thus, it is likely that the difference in glutamate levels between this study and our previous MRS study may be due to the difference between CSF samples and specific corticolimbic areas. Second, here, we specifically measured glutamate separate from glutamine, while in the MRS study, for technical issues, we assessed levels of Glx, namely, the sum of glutamate and glutamine. Third, although it is still not clear whether the low concentration of glutamate in CSF reflects a primary neuronal number or synaptic deficit or whether it represents a secondary compensatory adaptation, we might hypothesize that different areas of the brain, including the limbic system and other areas known to be involved in the pathophysiology of FMD, such as the dorsolateral prefrontal cortex, might abnormally express NMDA-R.

This study has the following limitations: first, the small sample size might limit the generalizability of our results; second, given that previous studies have shown that a good proportion of patients with FMD present psychiatric comorbidities, the absence of psychiatric comorbidities in our sample might limit the generalizability of our results; third, the heterogeneity of controls might also limit our findings. However, to the best of our knowledge, no previous study has ever shown increased CSF glutamate levels in patients with Wernicke encephalopathy, vascular encephalopathy, and Tolosa–Hunt syndrome. With respect to multiple sclerosis, although glutamate seems to be implicated in its pathogenesis, a recent study showed that the levels of CSF glutamate in patients with multiple sclerosis did not differ from those in healthy controls^13^. Moreover, our data are substantially in line with the results of previous studies assessing levels of CSF glutamate with the same methodology in healthy subjects (normal range 0.6–3 μmol/L)^8^. Concerning this aspect, it is worthwhile to note that although the means of CSF glutamate levels of both patients with FMD and controls fall within this normal range, the range appears to be very wide, and the difference between the two groups, despite the small sample size, is statistically significant.

In conclusion, our findings argue in favor of glutamatergic dysfunction as a possible biological marker in the pathophysiology of FMD. Moreover, our results might have some therapeutic implications in terms of testing novel pharmacological approaches with drugs acting on NMDA-R in FMD.

## Methods

### Procedure

This was a retrospective study, including all FMD patients whose CSF was stored at −80 °C and registered in the laboratory information system of three Italian university hospitals in Milano (San Paolo Hospital, Policlinico Hospital, and Carlo Besta Neurological Institute) between June 2017 and December 2019. CSF samples were collected in the same way in each center: they were collected into 15 mL polypropylene tubes by LP in the L3/L4 or L4/L5 interspace. The LP was carried out between 8 and 10 a.m. after one-night fasting. Following LP, CSF samples were centrifuged at 2000 r/min for 10 min at 4 °C. The supernatants were aliquoted in polypropylene tubes and stored at −80 °C until use.

Through a review of patient charts, we registered age, gender, major psychiatric comorbidity (assessed through the clinical interview Structured Clinical Interview for DSM-5)^14^, the severity of FMD according to the Psychogenic Movement Disorders Rating Scale (PMDRS) score^15^, date of symptom onset, date of LP, sample arrival date at the laboratory, and date and result of CSF measurements. Controls were patients who had LP for diagnostic purposes other than FMD. The CSF analysis (along with other investigations) in controls led to the following conclusions: the absence of neurological disorders in three subjects, Wernicke encephalopathy in two subjects, multiple sclerosis in two subjects, vascular encephalopathy in one subject, and Tolosa–Hunt syndrome in one subject. Patients with FMD were included if they had “clinically established and documented” FMD according to the Fahn and Williams criteria^16^. Although participants had CSF collected for diagnostic purposes, they all signed written informed consent for the possible use of their CSF for scientific research purposes. The study was approved by the ethics committee of ASST Santi Paolo e Carlo (Milan, Italy).

CSF measurements were performed in a single run on March 2020 at the Istituto di Ricerche Farmacologiche Mario Negri IRCCS in Milano. Amino acids were assayed by high-performance liquid chromatography coupled to fluorimetric detection as previously described, with minor modifications^17^. Briefly, 100 μL of CH_3_OH was added to 100 μL of thawed sample, vortexed for 20 s, and centrifuged (10,000 × *g* for 10 min) at 4 °C. Fifty microliters of supernatant was diluted with 200 μL borate buffer (0.1 M, pH 9.3), and 50 μL of this solution was transferred to the autosampler mini vial. Samples were derivatized with *o*-*phthalaldehyde*-mercaptoethanol reagent according to a previously described procedure^17^. Amino acid levels were expressed in μmol/L

### Statistical analysis

The Kolmogorov–Smirnov test was performed to assess whether the continuous variables respected the assumption of a normal distribution. The differences between patients with FMD and controls for the variable age, PMDRS score, and time elapsed between date of the LP and date of CSF amino acid measurements were calculated using standard *t* tests. To assess the differences between patients with FMD and controls for the levels of CSF amino acids, a multivariate ANOVA with glutamate, glutamine, alanine, and asparagine as dependent variables and “Group” as a factor was run. Categorical variables were analyzed using the Pearson’s *χ*^2^ test. Spearman’s correlation analyses were run between amino acid levels and (i) severity of FMD and (ii) duration of disease.

## Supplementary information

NPJPARKD-00462R_nr-reporting-summary

## Data Availability

The data that support the findings of this study are available from the corresponding author upon reasonable request.
